# Supplementary material for: A Kirchhoff-Nernst-Planck framework for modeling large scale extracellular electrodiffusion surrounding morphologically detailed neurons
Source: PLoS Comput Biol. 2018 Oct 4;14(10):e1006510. doi: 10.1371/journal.pcbi.1006510 (PMC6191143; doi:10.1371/journal.pcbi.1006510)
Supplement: S1 Appendix — (PDF) [file pcbi.1006510.s002.pdf]

S2 Appendix: Details on the FEniCS implementation

The PNP- and KNP-schemes are represented by systems of nonlinear coupled equations, which must be solved numerically for all but the most trivial cases. In this study we solved the equations using the FEniCS software for finite element problems. FEniCS aims to provide automated computational mathematical modeling, in the sense that the user does not need to discretize the equations which are to be solved. Instead, the user provides the continuous form of the equations. FEniCS translates the problem into a discrete formulation, and solves the discrete problem using an appropriate solver. In this section we explain how to set up a solver for the KNP problem in FEniCS for a system consisting of two ions with opposite charge.

Time discretization

We use the superscript n to denote that a variable is evaluated at the time step n. The corresponding time is $t^n = n\Delta t$. As an example, c_k^n denotes the concentration of ion species k at time step n. The Nernst-Planck equation takes the form

$$\frac{c_k^{n+1} - c_k^n}{\Delta t} = -\nabla \cdot J_k^{n+1} + f_k^{n+1}, \tag{1}$$

where

$$J_k^{n+1} = -\left[D_k \nabla c_k^{n+1} + \frac{D_k F z_k}{\psi} \nabla \phi^{n+1}\right]. \tag{2}$$

In both the PNP and KNP systems, the equations for the electric field do not contain a differentiation with respect to time, and so we evaluate all terms at the same time point. The equation is

$$\nabla \cdot (\sigma^{n+1} \nabla \phi^{n+1} + \nabla b^{n+1}) + F \sum_k z_k f_k^{n+1} = 0, \tag{3}$$

for the KNP system.

Variational form of the PDE

The starting point is to express the KNP equations in variational form. To do this, we multiply the PDE by a *test function* v and integrate over the domain Ω. The variational form of the Nernst-Planck equation is

$$\int_\Omega \left(c_k^{n+1} - c_k^n - \Delta t f_k^{n+1}\right) v \,\mathrm{d}x + \Delta t \int_\Omega (\nabla \cdot J_k^{n+1}) v \,\mathrm{d}x = 0. \tag{4}$$

We can improve the efficiency of the solver by lowering the order of the spatial derivative. Integration by parts on the second term gives

$$\int_\Omega \left(c_k^{n+1} - c_k^n - \Delta t f_k^{n+1}\right) v \,\mathrm{d}x + \Delta t \int_{\partial\Omega} (J_k^{n+1} \cdot \mathbf{n}) v \,\mathrm{d}s - \Delta t \int_\Omega J_k^{n+1} \cdot \nabla v \,\mathrm{d}x = 0, \tag{5}$$

where the second integral is along the boundary of the domain, and $\mathbf{n}$ is a normal vector pointing out of the domain. For the case of the no-flow boundary,

$$J_k^{n+1} \cdot \mathbf{n} = 0, \tag{6}$$

and for the case of the concentration-clamp boundary condition, the test functions v are chosen such that they are zero on the boundary. In both cases, the boundary integral in Eq. (5) evaluates to zero.

A similar consideration for the electroneutrality equation in the KNP system, gives the variational form

$$\int_\Omega (\sigma \nabla \phi + \nabla b) \cdot \nabla v \, \mathrm{d}x - \int_{\partial\Omega} (\sigma \nabla \phi + \nabla b) \cdot \mathbf{n} v \, \mathrm{d}s + F \int_\Omega \sum_k z_k f_k^{n+1} v \, \mathrm{d}x = 0, \tag{7}$$

where the boundary integral evaluates to zero, due to the boundary conditions.

FEniCS implementation

To illustrate how the PDEs are implemented in FEniCS, present a tutorial problem, also found at the FEniCS website (`https://fenicsproject.org`), showing how to implement the Poisson equation with a fixed boundary,

$$\nabla^2 \phi = f, \qquad \text{in } \Omega, \tag{8}$$

$$\phi = g, \qquad \text{at } \partial\Omega. \tag{9}$$

The first step, assuming that the computational mesh is known, is to define the function space.

```
# Define function space
P1 = FiniteElement('P', interval, 1)
V = FunctionSpace(mesh, P1)
```

The PDE is transcribed into code form by using mathematical operators in FEniCS.

```
# Define variational problem
u = TrialFunction(V)
v = TestFunction(V)
a = inner(grad(u), grad(v))*dx
L = f*v*dx
```

The boundary condition must be defined.

```
bc = DirichletBC(V, g, "on_boundary")
```

Finally, the solution is computed.

```
# Compute solution
w = Function(W) # solution will be stored to w
solve(a == L, w, bc)
```

The code for implementing the electrodiffusion models is more comprehensive, as the system of equations is more complicated. Including the complete code here would be redundant as it is available online, at `https://github.com/CINPLA/KNPsim`.
